# Editorial Extracts and Gleanings

**Published:** 1874-01

**Authors:** R. C. Word

**Affiliations:** Georgia


By R. C. WORD, M.D., OF GEORGIA.

*Pancreatine.*—Some very interesting notes on this substance were read before a pharmaceutical meeting in Philadelphia in November last. The article has of late attracted considerable attention as a remedial agent, particularly in dyspeptic affections. We well remember the obscurity which prevailed in our early professional life with regard to the function of the pancreas; and it is surprising, even to this day, how few practitioners ever think of the existence of such an organ, much less its liability to disease.

We have long been of the opinion that most cases of dyspepsia, an affection which seems to be growing more and more common in this country of hasty and intemperate eaters, are due to disease, either functional or organic, of this viscus, or of the duodenum with which it is connected. About one-half the doctors, and nine-tenths of the quacks and nostrum vendors, saddle these cases upon the liver. The hog, an animal evidently constituted and designed by Nature to eat, digest and fatten, has been seldom, if ever, known to have a diseased pancreas ("sweet breads"), yet it is very common to find the liver of the hog extensively diseased with abscess and other lesions, and that, too, in most instances, without in the least affecting the animal's health, or retarding the fattening process.

Most of the so-called "liver medicines" which aid digestion are those that stimulate or excite the peristaltic action of the duodenum, into which the ducts from the liver and pancreas empty their secretions. Any purgative which acts upon the upper bowel thus assists digestion temporarily by encouraging or facilitating the flow of these secretions into the bowel. The *pancreatic fluid*, closely resembling saliva, *emulsifies* the oily and fatty globules contained in the food, and fits them for assimilation, and, we doubt not, is a more important agent in the process of digestion than bile. We think that many of our best antidyspeptic agents owe their good results to their action upon the duodenum and pancreas. We know that mercury acts specifically upon the salivary glands, which, in their anatomy as well as their secretion, resemble the pan creas in a most remarkable manner.

*A Test for Morphia.*—We learn from the American Journal of Pharmacy that if a substance containing morphia be heated gently with a few drops of sulphuric acid and a very small quantity of perchlorate of potassium, the liquid immediately surrounding the perchlorate will at once assume a deep brown color, which will soon spread and extend over a greater part of the acid. Warming increases the delicacy of the test. 0.0001 gramme of morphia can be distinctly recognized in this way, and no other alkaloid is acted upon in a similar way by these substances.

*How to Distinguish Sulphate of Morphine from Quinine.*—It not unfrequently occurs that the practitioner desires to distinguish between the sulphate of morphine and the sulphate of quinine. If a small quantity—say about a grain—of the sulphate of morphine be added to a teaspoonful of water, it will dissolve wholly, making a clear solution. A like quantity of quinine will not dissolve without the addition of an acid, but will make a cloudy or turbid mixture. This is simple, yet we have met physicians who did not know it.

*Ergot in Headache.*—Dr. Silver, of Ohio, says, in the Philadelphia Medical and Surgical Reporter, that no remedy will relieve a larger proportion of sick and nervous headaches than ergot. He thinks its *modus operandi* is by contracting the muscular fibers of the arterial walls, thus lessening the quantity of blood in the brain. Dose, ten to twenty drops of the fluid extract every half hour until relief follows.

*Pills of Sulphate of Quinia.*—Mr. C. C. Patterson, of St. Clairsville, Ohio, in a communication to the editor of the American Journal of Pharmacy, suggests that these pills may be made of small size, and without any gum, syrup or extract, by adding to the quinia a little tartaric acid and sufficient water. This is a modification of the plan published by Prosessor Parrish in that journal in 1853, and which consists in adding aromatic sulphuric acid to the quinia and rolling the mass before it hardens, which takes place rather suddenly, and may be retarded, particularly when operating upon a larger quantity, by the addition of a very small quantity of honey or simple syrup.

Quinine pills may be made very small by the use of syrup alone or molasses. It requires a very amall quantity of syrup. If the quantity of syrup be not too much, no other ingredient is necessary.

*Transfusion of Blood.*—An interesting paper on this subject has been translated from the German by Henry Kerchner, of St. Louis, Missouri. The operation is regarded as very simple. In case of need, any syringe, with a bistoury and forceps, will answer. The operation is old—was first tried on the dog by Lower in 1667. In 1824 James Bleandell first executed it on the human subject with human blood. He afterwards performed it three times successfully.

At a later period Dumas, Prevost, Banum, and others were active in the advocacy of transfusion; the last named gentleman, in a paper as late as 1863, showed scientifically that, for a man, only human blood should be used, and that *defibrinated.*

Transfusion is indicated in all those pathological conditions where the blood in quantity and quality is so altered that it is unfit to fullfil its functions. It is a justifiable operation in extreme cases of post portem hemorrhage, or in any case where life is in jeopardy from the loss of blood. The opinion is expressed that the time is not remote when this operation will become quite common, and that numerous lives will thereby be saved that otherwise would be lost.

Undefibrinated blood has been successfully used, but is not so safe on account of the tendency to clot, and the danger of embolism and thrombus. Defibrination is a simple work, and may be quickly executed. The writer thus describes this manipulation: "I have taken, in five transfusions made by me, a clean wooden stick for stirring the blood. After beating or stirring it for some minutes, I strained it through fine linen and then beat it anew. When I found no fibrin deposited on the stick I strained the second time, and now the blood was ready for injection. During the stirring, the vessel containing the blood is kept in another vessel with warm water of 38° C. temperature (100° F.). It is not necessary to be over anxious about one or two degrees. Even if the blood cools down somewhat it does not matter. This is in so far of importance to know, as in a country practice a thermometer is not always at hand to test the precise temperature." Though transfusion may in an emergency be performed with an ordinary syringe and knife, yet, when possible, it should be done with instruments specially made for the purpose. Access of air into the vessel must be carefully guarded against. The best instrument is the one invented by Uterhart. The quantity to be injected varies from 1 to 10 or 12 oz, according to the indication. In cases of ammonia it should be repeated several times, the quantity injected being small. The operation is described as follows:

"We should take example from Hiiter in our perseverance. Suppose we are called upon to make transfusion. A healthy, robust man, who is willing to lend his blood for the purpose, is at hand. We draw blood from a vein of his arm, and catch it in a clean vessel, and defibrinate it in the above stated manner. It is ready; you must keep it warm while you turn to the patient; you look for a favorably situated vein, say the median basilic or cephalic, which is laid bare by an incision. With a few more cuts you prepare it free in its circumference, the distal end is now ligated, so as to keep the field of operation free from blood. A little above you place a second ligature, but without tightening it. You now fill a Uterhart's syringe and also its canula, and carry the latter through the incision into the vein. To the canula you attach the syringe and drive, with a slow rotary movement of the piston, the blood into the vein. Some persons you will find to whom you can entrust the pulse of the other side and who counts its beats aloud. In doing this we are secure against accidents, and you can see the breathing of the *not chloroformed* patient during the injection yourself. A slight deviation of the pulse always occurs; it either becomes more full, or it becomes for a moment smaller. This need not disturb the operator; so a slight cough, or disquietude of the patient. Strong oppression of the chest, irregularity of the pulse, demand caution, but syncope demands discontinuance of the operation. You will certainly guard against some of these eventualities by quietly talking to the patient, for the moral impression of the operation is a great one. Accidents demanding a discontinuation of the operation are very rare. I only would not leave them unmentioned. When the injection is completed you take out the canula, tie the vein above, and cut it through between the two ligatures, and sew up the wound, one turn of the roller bandage completes the whole simple manipulation.

It is known that the intra-uterine injection of a solution of the perchloride of iron will almost infallibly arrest post-partum hemorrhage, even in cases where the contraction of the uterus cannot be accomplished. Yet, from a discussion which we note in *The Obstetrical Society of London*, it would seem that the use of this agent is sometimes followed by *puer-peral peritonites.* Dr. Sell, however, of New York, said, "That his experience regarding the use of perchloride iron was obtained at the University of Vienna, which could boast of from 7,000 to 9,000 deliveries annually. There its use in post-partum hemorrhage was the treatment upon which they relied, provided ergot and injection of cold water did not arrest the bleeding. A weak solution of the ferrum sesquichloridum (ʒj ad aq. lb. j.) was gently injected, and repeated till the hemorrhage ceased He had never seen any bad results from this treatment."

Dr. J. J. Phillips, while admitting that there were certain dangers connected with the injection of a solution of perchloride of iron, believed there was no valid argument against its use in suitable cases. He had used it several times and death had occurred only in one case, which he could not in the least degree connect with the use of the iron. He generally diluted the liquor ferri perchloridi (not the strong one) with about half its bulk of water.

Dr Playfair said that he should much regret if the case brought before the Society should have the effect of throwing doubt on the safety of astringent injections in severe cases of post-partum hemorrhage. He had used the perchloride of iron in many cases, and only once unsuccessfully; nor had he ever seen any evil consequences. Dr. Heywood Smith's case was one of secondary hemorrhage caused by the presence of a piece of retained placenta; and the strong undiluted liquor ferri perchloridi had been injected—a proceeding which Dr. Barnes had not sanctioned.—*Virginia Clinical Record.*

